# Safety of Postpartum Herbal Medicine in Primary Care Korean Medicine Clinics: Protocol for a Prospective, Multicenter, Registry-Based Observational Study

**DOI:** 10.2196/87543

**Published:** 2026-04-17

**Authors:** Anna Kim, Eunbyul Cho, Young-Eun Kim, Sumin Seo, Sungha Kim, Jae-Ahn Shin, Eunhee Lee, Mi Ju Son

**Affiliations:** 1 Korea Institute of Oriental Medicine Daejeon Republic of Korea; 2 Wonkwang University Iksan Republic of Korea; 3 Jayoon Korean Medicine Clinic Bucheon Republic of Korea; 4 Woosuk University Korean Medicine Hospital Jeonju Republic of Korea

**Keywords:** postpartum period, herbal medicine, safety, adverse events, registry

## Abstract

**Background:**

Postpartum conditions significantly affect maternal and infant health. In South Korea, herbal medicine is commonly used for postpartum care in Korean medicine, but safety data remain limited. This study outlines a multicenter Korean medicine clinic registry designed to investigate treatment patterns and adverse events (AEs) associated with postpartum herbal medicine use. This primary care, practice-based research network registry will inform clinical decision-making and pharmacovigilance in postpartum care, supporting the quality and safety of care and guidance for comedication monitoring across community clinics.

**Objective:**

This study aims to establish a multicenter registry to investigate AEs and treatment patterns associated with postpartum herbal medicine use in Korean medicine clinics.

**Methods:**

This prospective observational registry will enroll postpartum patients receiving herbal medicine treatment from 15 Korean medicine clinics between July 2024 and June 2027, with a target sample size of 1000 participants. Patients within 6 months postpartum receiving herbal medicine will be included. Data will be collected for up to 8 weeks, including demographic information, medical history, herbal medicine prescriptions, AEs, and concomitant medication use. AEs are self-reported by patients, and causality is assessed using the World Health Organization Uppsala Monitoring Centre system and the Naranjo algorithm. Data collection and entry will follow standard operating procedures.

**Results:**

The registry was registered with the Clinical Research Information Service in June 2024 (KCT0009552). Recruitment began in July 2024 across 15 Korean medicine clinics and is planned to continue until June 2027 with a target enrollment of 1000 postpartum patients.

**Conclusions:**

This study represents the first large-scale, multicenter registry examining herbal medicine use during the postpartum period in Korean medicine clinics. By systematically collecting real-world data on AEs associated with postpartum herbal medicine use, we aim to establish a comprehensive safety database for individual herbal medicine and constituent herbs. The findings may support pharmacovigilance of herbal medicine and improve postpartum care outcomes for mothers and infants.

**Trial Registration:**

WHO ICTRP KCT0009552; https://trialsearch.who.int/?TrialID=KCT0009552

**International Registered Report Identifier (IRRID):**

DERR1-10.2196/87543

## Introduction

### Background

Childbirth, although a natural and universal process, imposes considerable stress on the mother’s body, often resulting in a debilitated condition and a low quality of life [1,2]. Studies show that 49% to 94% of women experience at least 1 health issue up to 8 weeks post partum [3,4]. These issues include fatigue [5-9], headache [10-12], musculoskeletal pain [8,13,14], urinary incontinence [15-17], and depression [18-21], often persisting beyond the first year post partum. These prevalent and prolonged health problems significantly affect the well-being of both mothers and their children [22]. This situation highlights the need for safe and effective postpartum care strategies, leading many to consider herbal medicine as a treatment option [23,24].

Herbal medicine offers various potential benefits for postpartum women. Studies have shown that can mitigate postpartum pain [25]. Additionally, when used in conjunction with conventional treatments, herbal medicine may alleviate postpartum depression [26]. In East Asian countries, where herbal medicine is integrated into national health care systems, its use in postpartum care is particularly prevalent, with high satisfaction among consumers. Nevertheless, the lack of safety data on herbal medicine for both mothers and infants remains a considerable concern, possibly deterring some from using herbal medicine [23,27]. For instance, a study in Taiwan found that breastfeeding mothers frequently used HM; however, those consuming traditional Chinese herbs had elevated lead levels in their breast milk, emphasizing the importance of safety monitoring [28]. This global context underscores the urgent need for rigorous safety studies on postpartum herbal medicine use. In Korean medicine practice, postpartum herbal treatment often includes individualized prescriptions derived from traditional formulations such as *Boheo-tang, Ssanghwa-tang, Gunggwijohyeol-eum,* and *Dangguijakyak-san*, which are commonly used to address postpartum symptoms, including fatigue, musculoskeletal pain, and circulatory disturbances. Recent studies have reported that herbal medicine interventions may improve pain, quality of life, and depressive symptoms in postpartum women while demonstrating acceptable safety profiles [25,29,30].

In South Korea, Korean medicine plays an active role in addressing postpartum symptoms, with herbal medicine commonly used alongside acupuncture as the standard treatment method. According to a study on the satisfaction of mothers who received postpartum treatment with Korean medicine during the early postpartum period, 78.68% indicated a willingness to use Korean medicine again in future childbirths [31], while satisfaction with herbal medicine treatment was notably high at 72.13% [32]. Numerous local governments in South Korea support the use of herbal medicine after childbirth by subsidizing expenses, further indicating its perceived value in postpartum care [32].

However, South Korea’s drug use review service does not provide adverse event (AE) information related to herbal medicine. While some national statistics provide estimates on the incidence rate of AEs related to herbal medicine, these figures are based on patient reports or survey data, lacking expert case evaluations and detailed information on specific herbal medicine treatments [33]. Moreover, some reported AEs may not be directly attributable to herbal medicine, underscoring the importance of assessing a clear causal relationship [34]. This lack of comprehensive safety data creates a significant gap in our understanding of the risks associated with postpartum herbal medicine use.

To address these concerns, especially the lack of safety information from official sources, this study aims to establish a registry for those receiving herbal medicine treatment at primary care institutions, specifically Korean medicine clinics. Because Korean medicine clinics account for approximately 96% of Korean medicine institutions in South Korea [35], research based on primary care Korean medicine clinics is crucial for collecting representative clinical data on Korean medicine. Through the systematic collection of data on the constituents of herbal medicine, details of AEs, and causality assessment, we seek to provide evidence for the safety monitoring of herbal medicine administration during the postpartum period.

The primary research questions of this study are as follows: (1) What is the incidence rate of AEs following the use of postpartum herbal medicine? (2) Which herbal medicines and constituent herbs are involved in reported AE cases? (3) What is the causality classification of the observed AEs?

This study represents the first nationwide multicenter registry examining the use of herbal medicine during the postpartum period in Korean medicine clinics across South Korea. By providing comprehensive safety data, this research aims to contribute to evidence-based clinical practice in South Korea and ultimately enhance patient safety for both mothers and infants.

### Study Objective

The objective of the Safety of Herbal Medicine Registry in Korean medicine clinics (SAFEHERE-KM) is to systematically collect and analyze clinical data on AEs related to herbal medicine treatment in postpartum patients in South Korea. This web-based registry will collect patient characteristics, details of herbal medicine prescriptions, and AE information, including a causality assessment of any AEs that arise during herbal medicine treatment using the World Health Organization Uppsala Monitoring Centre causality assessment [36] and the Naranjo algorithm score [37,38]. By establishing AE monitoring databases, this registry further aims to contribute to the development of an active pharmacovigilance system for herbal medicine in South Korea.

## Methods

### Study Design

The SAFEHERE-KM registry is a multicenter, prospective, observational study designed to collect data on routine clinical care and associated AEs in postpartum women receiving herbal medicine treatment at Korean medicine clinics. This study aims to enroll at least 1000 eligible patients between July 2024 and June 2027 based on a previous study suggesting that this sample size rapidly decreases variation when reporting AE incidence [39]. Using empirical observations of postpartum patient visits to participating Korean medicine clinics, we anticipate enrolling approximately 400 participants annually (approximately 27 patients per clinic per year).

The participants are postpartum patients who voluntarily consent to the collection of their clinical data. This study does not introduce specific interventions. Instead, the patients receive individualized herbal medicine treatments as part of their routine postpartum care. Korean medicine doctors prescribe herbal medicine at their discretion without restrictions. This registry focuses on AEs experienced by postpartum women receiving herbal medicine, and infant AEs are not systematically assessed because infants are not enrolled as study participants.

Throughout the treatment period, which may extend up to 8 weeks depending on the duration of prescribed herbal medicine use, we collect data on demographics, medical history, Korean medicine treatment details, herbal medicine prescriptions, AEs, and concomitant medication use. AEs are self-reported by patients via an online survey and recorded in online databases. AEs, including the absence of an AE, will be recorded through scheduled assessments approximately every 2 weeks during the herbal medicine treatment period, with follow-up continuing for up to 8 weeks. The electronic case report form includes predefined fields to record AEs and to indicate whether an event meets the criteria for a serious AE. To ensure consistency in data collection and entry, Korean medicine doctors follow standard operating procedures specifically designed for this registry. All reported AEs, including serious AEs, are reviewed and validated by licensed Korean medicine doctors.

Patients will be recruited from 15 Jayoon Korean medicine clinics, a network specializing in gynecological disorders, located across South Korea. This network provides wide geographical coverage, including 5 clinics each in Seoul and Gyeonggi-do, as well as clinics in Jeju Province, Busan, Incheon, Daejeon, and Daegu (Multimedia Appendix 1).

The registry was registered in the Clinical Research Information Service (KCT0009552). Recruitment started on July 7, 2024.

### Eligibility Criteria

This registry enrolls patients diagnosed with complications predominantly related to the puerperium (O85-O92 of the International Statistical Classification of Diseases-10) or puerperal wind disorder (U32.7 of the Korean Standard Classification of Diseases-8). Puerperal wind disorder indicates a set of symptoms including pain and autonomic disturbance caused by inadequate body care after childbirth [1]. Women who visited the primary Korean medicine clinics within 6 months after giving birth and have been prescribed herbal medicine for postpartum recovery are included. The inclusion and exclusion criteria are presented in Textbox 1.

**Inclusion and exclusion criteria.**

**Inclusion criteria**

- Women aged ≥19 years
- Have visited primary Korean medicine clinics within 6 months post partum (calculated from the date of delivery) and are undergoing herbal medicine treatment for postpartum recovery

**Exclusion criteria**

- Did not consent to participate
- Deemed inappropriate for participation in this study

### Recruitment

Participants will be enrolled in 15 primary Korean medicine clinics that offer postpartum care. Recruitment posters will be placed at the entrance of each clinic to encourage voluntary participation. Health care professionals will explain the study’s purpose and procedures—including data use, confidentiality, and data sharing for analysis—and obtain written informed consent from eligible participants before data collection. Participants may withdraw from the study at any time without any impact on their clinical care. Recruitment began in July 2024 and is currently ongoing across all 15 Korean medicine clinics.

### Study Outcomes

For participants who meet the inclusion criteria and voluntarily sign the written informed consent form, we will assign a study registration number. Subsequently, we will collect data for up to 8 weeks while they consume herbal medicines (Textbox 2).

Table 1 presents the details of the data collection and the study schedule.

**Data collected for up to 8 weeks while postpartum patients consume herbal medicines.**

- Demographics: date of birth and age
- Anthropometric measurements: height and weight
- Medical history (maternal health and delivery details): delivery information (date and mode of delivery and gestational age); obstetric history (number of pregnancies, full-term births, preterm births, miscarriages, and surviving children); high-risk pregnancy factors (advanced maternal age [≥35 years], in vitro fertilization, gestational hypertension, and gestational diabetes); and newborn information (demographics, birth details, and feeding methods)
- Details of herbal medicine prescription: name of prescribed herbal medicine, start date of administration, composition (herbs and doses), total and 1-day dose of herbal medicine, and duration of herbal medicine administration
- Other Korean medicine treatments: concurrent treatments, such as acupuncture, moxibustion, cupping, Chuna manipulation, and Korean medicine physical therapy
- Details of adverse events (AEs): the occurrence of AEs is monitored at each visit during herbal medicine intake for up to 8 weeks. If an AE occurs, the collected data include the AE start date, symptoms, outcome, severity, actions taken, recurrence after the reintroduction of the suspected herbal medicine, treatment methods for the AE, and concurrent medications. Follow-up telephone interviews may be conducted for detailed information and causality assessments.

**Table 1:** Study schedule.

Time points			Visit 1: baseline^a^		Subsequent visits at 2, 4, 6, and 8 weeks after taking herbal medicine^b,c^		End point: follow-up completion^a,d^
Informed consent			✓				
Demographics			✓				
Diagnosis			✓				
Eligibility screening			✓				
Anthropometrics			✓				
Medical history			✓				
Concomitant medication			✓		✓		✓
**Korean medicine treatment information**							
	Details of the herbal medicine prescription					✓	
	Other Korean medicine treatments					✓	
**Check for adverse events**							
	Occurrence status			✓		✓	
	Details of adverse events^e^			✓		✓	

^a^Data collected from the Korean medicine doctor.

^b^Visits 3, 4, and 5 may be conducted if herbal medicine intake continues, with the process occurring at 2-week intervals for up to 8 weeks.

^c^Data collected from the patient.

^d^Follow-up completion is conducted once data collection of adverse events is finalized or when herbal medicine administration is completed.

^e^Data are collected only in the presence of adverse events. Adverse events represent the primary outcome of this registry.

### Data Management and Quality Control

For data collection, we will use the myTrial Electronic Data Capture system (NIKOM), an electronic case report form system validated by the National Agency for Korean Medicine Innovative Technologies Development. Each clinic’s Korean medicine doctor can access the data entry form via the web server from a remote computer with internet access by logging in using the credentials provided by the website administrator [40]. Each case entered into the registry will be reviewed regularly, and data queries will be generated according to the data monitoring manual. Monitors regularly visit Korean medicine clinics to check the original registry records and research files, and they discuss and resolve any issues with the coinvestigators and principal investigators at each institution. All data collected for this study will be securely stored on a dedicated server with strict access-control permissions.

### Statistical Analysis and Causality Assessment

Statistical analyses will be performed using R (version 4.5.0; R Foundation for Statistical Computing). For participants who withdraw consent for continued follow-up, the data collected until withdrawal will be used in the analysis. For continuous variables, the means and SDs will be presented. Categorical variables will be reported as frequencies and percentages. Herbal medicine exposure will be categorized based on high-frequency prescriptions, major prescription groups, and functional classifications. Patterns of herbal medicine use in AE cases will be explored descriptively. The incidence of AEs will be presented as the proportion of participants experiencing at least 1 AE, and all AEs will be summarized using descriptive statistics. In addition, for cases in which AEs occur, the corresponding prescribed herbal medicine and their constituent herbs will be summarized using frequencies and proportions to describe observed patterns.

For any reported AEs, severity will be determined based on established criteria for assessing AE severity [41]. These criteria are commonly used in clinical safety studies to evaluate the severity of AEs and are applicable to general clinical symptoms regardless of treatment modality. The causality of AEs will be evaluated using both the World Health Organization Uppsala Monitoring Centre causality assessment [36] and the Naranjo algorithm score [37,38], based on the approach used in similar previous studies [42,43]. This evaluation will be carried out by an independent Adverse Reaction Assessment Committee affiliated with the Regional Drug Safety Center at the Korea Institute of Drug Safety and Risk Management.

### Patient and Public Involvement

Patients will not be involved in the design, conduct, and dissemination plans of this research, as this registry study aims to collect real-world data on routine clinical practice.

### Safety Considerations

While AE data are collected for research purposes, the treatment and management of any AEs are conducted by Korean medicine doctors as part of routine clinical care, ensuring that the participants receive appropriate care throughout the study period.

### Ethical Considerations

This registry protocol received approval (version 1.0; April 24, 2024) from the institutional review board of Woosuk Korean medicine Hospital, Woosuk University, Jeonju, Republic of Korea (H2404-01-01). Following amendments to the research plan, the updated protocol (version 1.1) was subsequently approved on June 24, 2024. Any protocol amendments will be approved by the institutional review board and communicated to all investigators. The findings will be shared through publication in peer-reviewed journals and presentations at academic conferences. Written informed consent will be obtained from all participants prior to enrollment. Participant data will be recorded in case report forms and stored in secure databases with restricted access to ensure privacy and confidentiality. Participants will not receive financial compensation for participation in this registry.

## Results

The SAFEHERE-KM registry was registered with the Clinical Research Information Service on June 20, 2024 (KCT0009552) [44]. Recruitment began on July 7, 2024, across 15 participating Korean medicine clinics. Enrollment is planned to continue until June 2027 with a target sample size of 1000 postpartum patients.

## Discussion

The SAFEHERE-KM registry is expected to generate comprehensive real-world safety data on herbal medicine use during the postpartum period in Korean medicine clinics. By systematically collecting AE information and treatment details, this registry aims to provide an evidence base for monitoring the safety of postpartum herbal medicine use and supporting pharmacovigilance in primary care settings.

The SAFEHERE-KM study is the first large-scale, multicenter registry specifically designed to assess the safety of herbal medicine use during the postpartum period in primary Korean medicine clinics. This unique approach allows for the collection of real-world data on herbal medicine use in a setting where it is most commonly prescribed, potentially providing more representative and clinically relevant information than our previous study conducted in a Korean medicine hospital [45].

The puerperal period commonly extends beyond the initial 6 to 8 weeks when reproductive organ recovery is mostly complete. Many women continue to experience physical and mental burden because of childcare, household responsibilities, and stress. Insufficient care during this extended period can lead to various complications [1]. A previous survey has shown that mothers typically seek herbal medicine treatment at Korean medicine institutions approximately 13 to 14 weeks post partum. Recognizing the need for prolonged health management, many local governments in Korea provide support for Korean medicine treatments for up to 6 months post partum [32]. Therefore, we include women within 6 months of delivery in our registry. The decision to monitor AEs for up to 8 weeks after the initiation of herbal medicine treatment was based on clinical experience from participating Korean medicine doctors, who reported that most patients complete their herbal medicine treatments within this period. This approach allows us to maximize participant enrollment within our budget constraints while capturing the most relevant period of herbal medicine use in postpartum care.

The anticipated results of this study have several important clinical implications. By systematically collecting data on AEs associated with postpartum herbal medicine use, we aim to establish a comprehensive safety database for individual herbal medicine and constituent herbs. This dataset will include signs, symptoms, frequency, and severity of AEs, thereby providing valuable insights for both practitioners and patients. Furthermore, the causality assessment of reported AEs will strengthen the evidence for herbal medicine safety, promoting the development of guidelines for herbal medicine monitoring and decision-making processes in postpartum care. A dataset on causal herbs, their dosages, and concomitant drugs will enable the advanced analysis of herb-herb and herb-drug interactions [46].

Methodologically, the SAFEHERE-KM study has several strengths. The use of standardized data collection procedures, including detailed standard operating procedures and an electronic case report form, ensures consistency and quality in data gathering across multiple sites. This study collects closely monitored, patient-reported AEs to prevent underreporting and collect patients’ viewpoints [47]. We will recruit at least 1000 patients to gain an in-depth understanding of the AEs associated with herbal medicine. Patients are being recruited from 15 geographically diverse Korean medicine clinics across South Korea to minimize selection bias. Moreover, through data collection in real-world clinical settings across multiple primary care Korean medicine clinics, our results may achieve high external validity, making the findings more applicable to clinical practice [48].

Nevertheless, some limitations of this study should be acknowledged. The recruitment of participants from a single network of Korean medicine clinics that specialize in gynecological disorders may limit the diversity of herbal medicine prescriptions. While Korean medicine doctors in this network prescribe personalized herbal medicine for individual patients instead of uniform prescriptions, this may not capture the full range of herbal medicine practices across South Korea. Additionally, reliance on patient-reported AEs may underestimate unfavorable clinical events [49], although this is mitigated by standardized reporting procedures and visits to Korean medicine clinics during routine medical treatment. Because this registry primarily focuses on AEs associated with individual prescriptions, cumulative dose-response analyses based on total herbal medicine exposure may not be feasible.

Future research should build upon the foundation established by the SAFEHERE-KM study. The inclusion of a wider range of primary care institutions could provide more comprehensive evidence of herbal medicine use in postpartum care across various practice settings. Furthermore, long-term follow-up studies could offer insights into the long-term safety profile of postpartum herbal medicine use, which is beyond the scope of this study.

In conclusion, the SAFEHERE-KM registry is expected to provide important real-world evidence on the safety of postpartum herbal medicine use in Korean medicine clinics. This registry may contribute to improved pharmacovigilance and a better understanding of the safety profile of postpartum herbal medicine use. The findings of this study will contribute not only to the field of Korean medicine but also to global integrative approaches to postpartum care.

## Appendix

Multimedia Appendix 1 Locations of Korean medicine clinics participating in the registry.

## Abbreviations

AE: adverse event
SAFEHERE-KM: Safety of Herbal Medicine Registry in Korean medicine clinics

## Data Availability

Data sharing is not applicable to this study as no datasets were generated or analyzed during this study.
