# Supplementary figures and images for: Safety of Postpartum Herbal Medicine in Primary Care Korean Medicine Clinics: Protocol for a Prospective, Multicenter, Registry-Based Observational Study
Source: JMIR Res Protoc. 2026 Apr 17;15:e87543. doi: 10.2196/87543 (PMC13135165; doi:10.2196/87543)

## Slide 1
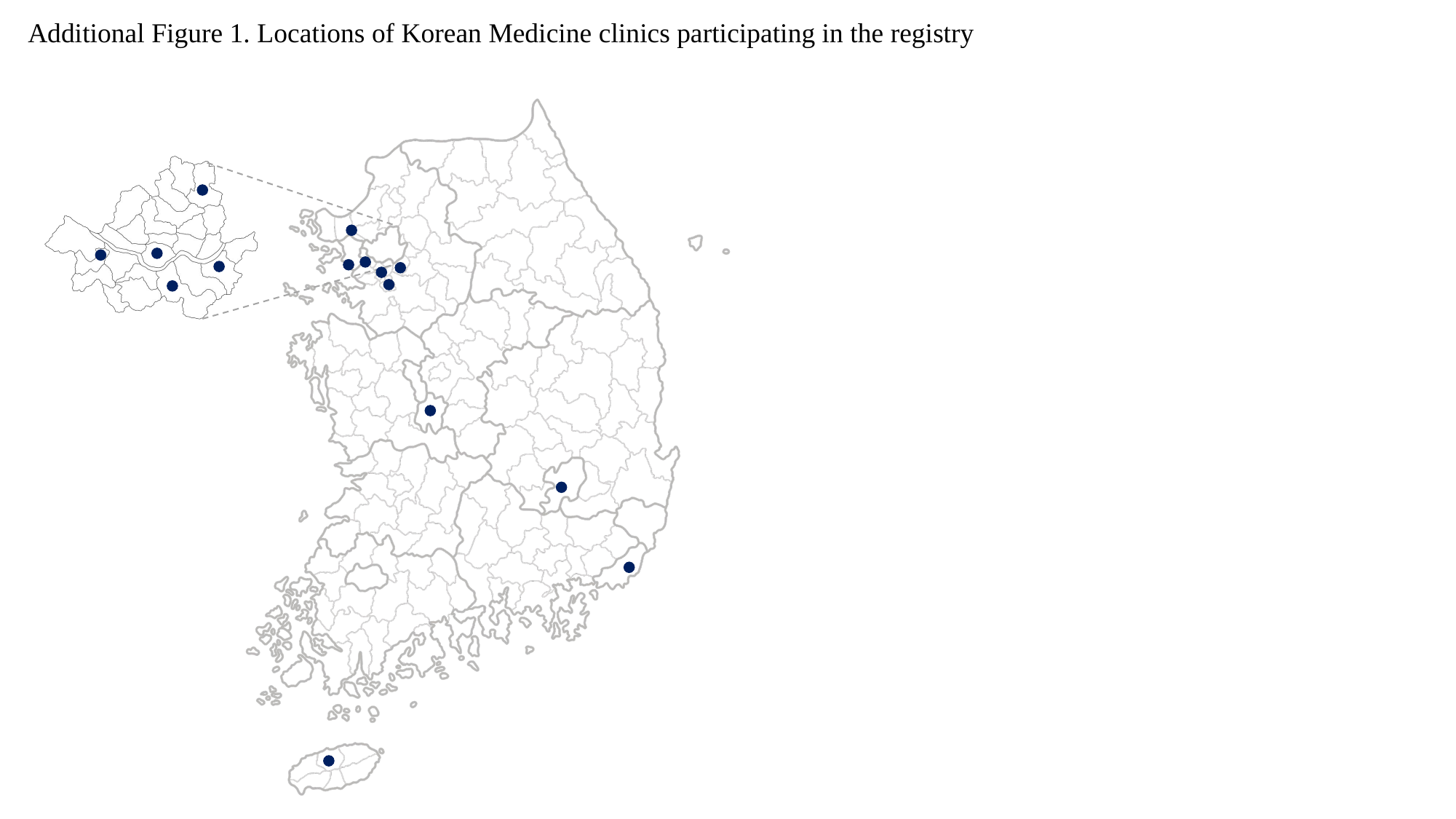

Additional Figure 1. Locations of Korean Medicine clinics participating in the registry

Supplement: Multimedia Appendix 1 [file resprot_v15i1e87543_app1.pptx]
